# Predicting Motor Imagery BCI Performance Based on EEG Microstate Analysis

**DOI:** 10.3390/brainsci13091288

**Published:** 2023-09-06

**Authors:** Yujie Cui, Songyun Xie, Yingxin Fu, Xinzhou Xie

**Affiliations:** 1 Shaanxi Joint International Research Center on Integrated Technique of Brain-Computer for Unmanned System, Northwestern Polytechnical University, Xi’an 710129, China; yujiecui@mail.nwpu.edu.cn (Y.C.); funancyx@163.com (Y.F.); xinzhxie@nwpu.edu.cn (X.X.); 2 Xi’an Aeronautics Computing Technique Research Institute, AVIC Xi’an, Xi’an 710068, China

**Keywords:** motor imagery, microstate analysis, subjects’ MI-BCI performance

## Abstract

Motor imagery (MI) electroencephalography (EEG) is natural and comfortable for controllers, and has become a research hotspot in the field of the brain–computer interface (BCI). Exploring the inter-subject MI-BCI performance variation is one of the fundamental problems in MI-BCI application. EEG microstates with high spatiotemporal resolution and multichannel information can represent brain cognitive function. In this paper, four EEG microstates (MS1, MS2, MS3, MS4) were used in the analysis of the differences in the subjects’ MI-BCI performance, and the four microstate feature parameters (the mean duration, the occurrences per second, the time coverage ratio, and the transition probability) were calculated. The correlation between the resting-state EEG microstate feature parameters and the subjects’ MI-BCI performance was measured. Based on the negative correlation of the occurrence of MS1 and the positive correlation of the mean duration of MS3, a resting-state microstate predictor was proposed. Twenty-eight subjects were recruited to participate in our MI experiments to assess the performance of our resting-state microstate predictor. The experimental results show that the average area under curve (AUC) value of our resting-state microstate predictor was 0.83, and increased by 17.9% compared with the spectral entropy predictor, representing that the microstate feature parameters can better fit the subjects’ MI-BCI performance than spectral entropy predictor. Moreover, the AUC of microstate predictor is higher than that of spectral entropy predictor at both the single-session level and average level. Overall, our resting-state microstate predictor can help MI-BCI researchers better select subjects, save time, and promote MI-BCI development.

## 1. Introduction

Electroencephalogram (EEG) devices are widely used to record brain signals in brain–computer interface (BCI) systems because of their non-invasiveness, high temporal resolution, and relatively low cost [1]. Motor imaginary (MI) is defined as the mental stimulation of particular limb movement (such as hands, feet, and tongue), and the execution of MI can evoke similar somatotopically organized activation patterns [2,3]. MI-based BCI requires no additional stimulation and is closer to “conscious” control, meaning that users imagine limb movements without actually moving the limb to control the system [4]. MI-BCI has become a research hotspot, and has broad applicability in the field of engineering and rehabilitation. Many studies about motor imagery have been reported in feature extraction [5,6], subjects’ MI-BCI performance [7], feedback experiments [4,8], and brain connectivity analysis [9].

Over the past few years, effective signal processing techniques have been applied to MI-BCI systems to extract informative and discriminative features [6]. Although these techniques have significantly improved MI-BCI control performance at the individual subject level, inter-subject variation remains a substantial problem. Many users cannot control BCI systems, and such users are described as demonstrating BCI inefficiency [10,11,12]. Some research has indicated that about 20% of people who attempt MI-BCI demonstrate such inefficiency [13]. It is necessary to develop methods to predict MI-BCI performance to effectively screen MI-BCI controllers. The resting-state EEG reflects the spontaneous activity of neurons, and has become the primary method for researchers to predict subjects’ MI-BCI performance. Blankertz et al. asserted that the good performer is likely to have a higher sensorimotor rhythms (SMR) potential during the resting state [14], which can be referred to as the rhythmic brain activities that decrease in the alpha (8–13 Hz) and beta (>30 Hz) frequency bands, and increase in the gamma frequency band. Ahn et al. reported that high theta (4–8 Hz) powers and low alpha powers during the resting state in the MI task can indicate BCI inefficiency [15]. Zhang et al. found spectral entropy derived from the eyes-closed resting-state EEG of channel C3 highly correlated with MI-BCI performance [16]. Lee et al. proposed a dynamic causal model to identify the connections of the resting-state network, which is able to predict MI performance [17]. Generally, the predictor of subjects’ MI-BCI performance is calculated from the resting-state EEG signal of a single channel (such as channel C3 or C4). Most of the research on MI-BCI was built with online processing conditions, so it only uses a single channel to reduce the dimensions of the data in order to achieve fast and efficient MI-BCI. However, using a single channel to predict subjects’ MI-BCI performance may lose spatial information. Some researchers have explored studies of MI-BCI by using multiple channels to fully use the spatial-temporal information of EEG. Lakshminarayanan et al. used nine electrodes across the SMR for discriminating tactile imagery across three digits in a single limb, which accomplished a higher classification performance [18]. Blanco-Mora et al. reported the performance of MI-BCI with different numbers of electrodes, and the results show that the more electrodes, the better the MI-BCI performance [19]. As a result, the improvement of MI-BCI performance benefited from using features from more electrodes than a single electrode. In addition, most studies only predicted subjects’ MI-BCI performance for a single session, and neglected cross-session performance analysis. Generally, the interval between each session can be longer in MI tasks, which may result in greater variability [20]. Therefore, it is necessary to explore the effects of applying these predictors across various sessions.

The alpha frequency band of the multichannel resting-state EEG signal can be parsed into a limited number of distinct quasi-stable states [21]. These discrete states, called ‘microstates’ are defined by topographies of electric potentials recorded in a multichannel array over the scalp, which remain stable for 80–120 ms before rapidly transitioning to a different microstate [22]. The EEG microstate analysis preserves the feature of both the temporal and spatial domains, and utilizes the multichannel information. Compared with other analysis methods of EEG, microstate analysis can observe millisecond changes in topographies. Besides, microstate transitions can represent rapid changes in brain activity that may underlie human cognitive function. Therefore, microstate analysis is able to research the differences between subjects. Using this method, brain electrical activity can be represented by a time series of microstates. At present, there are many research directions for EEG microstates, such as the theoretical basis of microstates [23], the connection between microstates and brain cognitive function [24,25], the application of microstate analysis in neuropsychiatric diseases [26,27,28,29], etc. Thus, microstates may capture more information on the resting-state EEG, providing a new measure to predict subjects’ MI-BCI performance.

In this paper, an EEG microstate analysis with multichannel signal was used in resting-state EEG, which would be meaningful to provide new insights for MI performance. The relationship between microstate time parameters and subjects’ MI-BCI performance was explored. Then, we used parameters with significant correlations to predict subjects’ MI-BCI performance, and compared the prediction performance with a spectral entropy predictor. The research aimed to prove that using the EEG microstate to investigate the spatial-temporal dynamics of inter-subject brain performance differences is feasible and effective.

The main contributions of this study are as follows:

- A microstate predictor was proposed to predict the performance of MI-BCI by using resting state data of EEG;
- We found two parameters of microstates that with the occurrence of MS1 and mean duration of MS3 can fit the performance of MI-BCI well;
- Our microstate predictor achieved a better prediction performance than the spectral entropy predictor;
- The EEG microstate analysis in this study provides an effective new way to analyze the differences in subjects’ MI-BCI performance.

The remainder of this paper is organized as follows. Section 2 presents EEG data description, data preprocessing, and the methods of EEG microstate analysis. Section 3 shows the experimental results. Consequently, Section 4 presents the discussion, and Section 5 presents the conclusions.

## 2. Materials and Methods

### 2.1. Data Description

**Figure 1:**
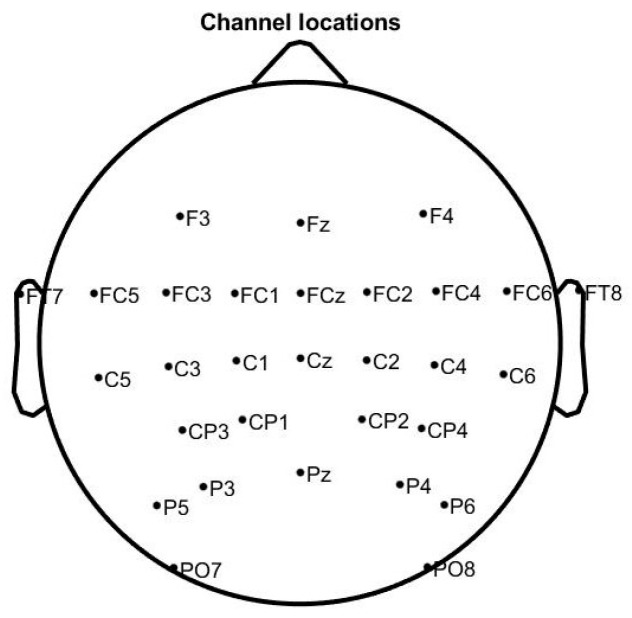
Electrode positioning.

Twenty-eight healthy subjects (mean age: 22.4 ± 3.7 years, male/female: 17/11) who reported no history of neurological diseases or psychiatric disorders were recruited to participate in the experiment. All subjects were right-handed with normal or correct-to-normal vision, and gave written informed consent. EEG signals were recorded using a Neuracle wireless amplifier from international 10–10 EEG caps with 64 electrodes located over the scalp region. The reference electrode was CPz, and the ground electrode was AFz. We selected 30 electrodes (F3, Fz, F4, FT7, FC5, FC3, FC1, FCz, FC2, FC4, FC6, FT8, C5, C3, C1, Cz, C2, C4, C6, CP3, CP1, CP2, CP4, P5, P3, Pz, P4, P6, PO7, PO8) for analysis, and the position of these electrodes is shown in Figure 1. We prevented the position of EEG electrodes varying between the sessions by measuring the center point of the head in both horizontal and vertical directions with the brain cap electrode when wearing the EEG cap.

**Figure 2:**
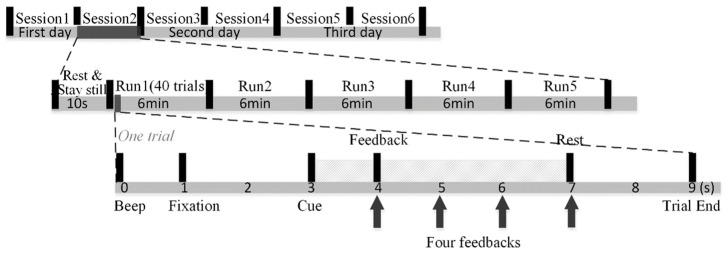
The experimental paradigm schematic diagram.

As shown in Figure 2, our MI experiments were conducted for three consecutive days. Each day comprised two sessions, leading to a total of six sessions and five runs, each of approximately a six-minute duration, making up one session. In each session, subjects were asked to rest and stay still for 10 s before the first run. Subjects could rest as long as they wished in the interval between the two runs. Each run is composed of 40 trials. At the trial start, a short 0.5 s beep sound with the frequency of 500 Hz prompted subjects to shift their attention to the screen. Then, the fixation appeared to remind subjects to rest and stay still. After two seconds, subjects performed motor imagery of the left hand when the left arrow appeared, or the right hand when the right arrow appeared. Note that we told subjects to keep their hands still while performing imaginary movements. The motor imagery task lasted four seconds. Afterwards, a 2 s rest period was offered, and the next trial followed. Furthermore, subjects were told they should suppress muscle movement to avoid artifacts. This paper used 1 s resting-state data before the left and right motor imagery tasks of all trials in dataset 3. All the experimental procedures were approved by the Northwestern Polytechnical University Medical and Experimental Animal Ethics Committee.

### 2.2. Data Preprocessing

The resting-state EEG signal was filtered between 7 and 30 Hz with a band-pass filter. Additionally, then, a blind source separation (BSS) algorithm was used to remove artifacts [30]. We realized the pre-processing of EEG data in the EEGLAB toolbox.

### 2.3. EEG Microstate Analysis

**Figure 3:**
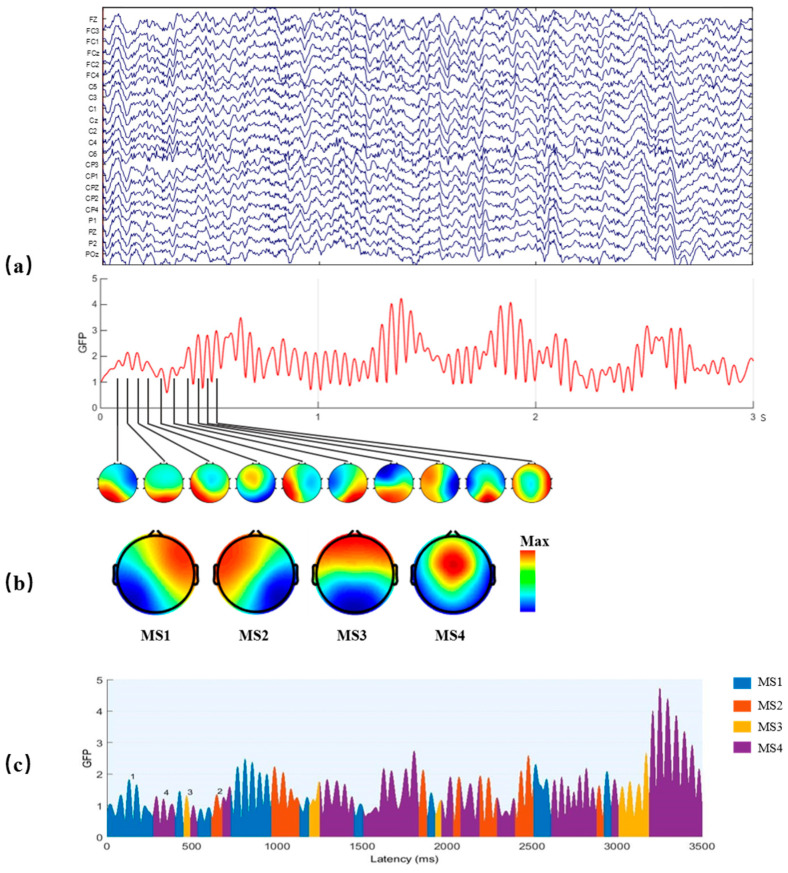
The method of EEG microstate analysis. (**a**) A multichannel EEG signal was used to calculate the GFP curve (drawn in red), and the peaks of the GFP curve were plotted to generate topographic maps. (**b**) These maps were clustered to four microstate maps (MS1, MS2, MS3, and MS4) using a modified K-means method based on topographic similarity. (**c**) Time series of microstates were obtained by fitting four classes microstates back to all data.

Global brain activity can be described by the global field power (GFP), which is the root of the mean of the squared potential differences at all N electrodes from the mean of instantaneous potentials across electrodes [31]. It is defined as follows:

(1) $$GFP(t)=\sqrt{\frac{1}{N}\cdot \sum_{i=1}^{N}(v_{i}(t)-\bar{v}(t){)}^{2}}$$

where $v_{i}(t)$ is the voltage at electrode i at time t, and $\bar{v}(t)$ is the average voltage across all electrodes at time t. The local maxima of the GFP curve represent instants of the strongest field strength and highest topographic signal-to-noise ratio (SNR), as shown in Figure 3a. Furthermore, the topographies of the electric field at the local maxima of the GFP curve are considered discrete states of the EEG, and the evolution of the signal is considered as a series of these states.

Based on GFP, a measure known as global map dissimilarity (GMD) of the spatial distance between two topographies can be defined as

(2) $${GMD}_{U,V}=\sqrt{\frac{1}{N}\sum_{i=1}^{N}(\frac{u_{i}-\bar{u}}{GFP_{u}}-\frac{v_{i}-\bar{v}}{GFP_{v}}{)}^{2}}$$

where $u_{i}$ and $v_{i}$ are the voltages of two topographies at electrode i, and $\bar{u}$ and $\bar{v}$ are the average voltage of two topographies across all electrodes. Therefore, GMD can be used to measure the spatial similarity. The closer GMD value is to 0, the more similar the two topographies are.

The original EEG signal may be represented by the topographies of the local maxima points of GFP, and these topographies were clustered by the modified k-means algorithm [32] to four microstate maps, labeled MS1-MS4, as shown in Figure 3b. The local maxima points of GFP mean finding the maximum GFP with 10 ms. After that, using the clustered four microstate maps as templates, all data were assigned to one of the four microstate classes, according to the minimal GMD of the four microstate maps. Then, the EEG signal was represented as a time series of alternating four microstate maps, also known as the time series of microstates, as shown in Figure 3c.

The time series of microstates provides a rich set of statistical parameters related to neurophysiology, and the following microstate time parameters were used as microstate feature parameters:

1. Mean duration: the mean length of time that one microstate keeps stable.
2. Occurrence per second: the number of times one microstate occurs per second.
3. Time coverage ratio: the ratio of the sum of the duration of one microstate to the duration of the EEG signal.
4. Transition probability (TP): Percent of transition from one microstate to another.

## 3. Results

The EEG microstate analysis was applied to the resting-state EEG data of 28 subjects. Then, based on the correlation between microstate feature parameters and the subject’s MI-BCI classification accuracy, a resting-state microstate predictor was proposed to predict the subjects’ MI-BCI performance effectively. The calculation of microstates and their feature parameters is in the Microstate EEGLAB toolbox. The Statistical Program for the Social Sciences (SPSS) (IBM SPSS Statistics v26.0) was used for statistical analysis.

### 3.1. MI-BCI Performance

The MI-BCI performance of all subjects was obtained from their classification accuracy. The EEG data of one session was recorded as a set of experimental data, and all the session data of 28 subjects were combined, resulting in a total of 168 sets of MI experimental data. A ten-fold cross-validation approach was chosen to evaluate the model’s performance. The feature extraction method adopted a common spatial pattern (CSP) [33], and support vector machine (SVM) was used as the classifier.

**Figure 4:**
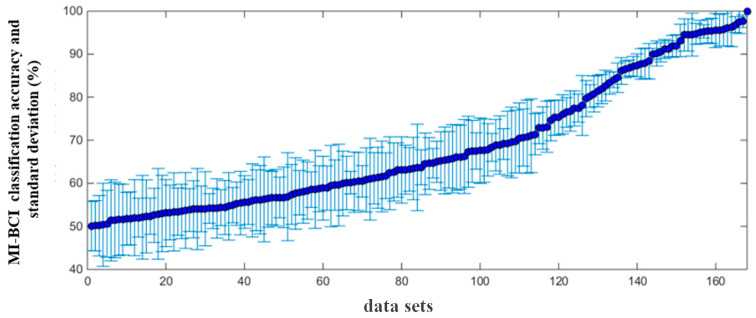
Distribution of the classification accuracy and standard deviation from 168 sets of MI data.

The MI-BCI classification accuracy and standard deviation calculated from 168 sets of MI experimental data are shown in Figure 4. The average classification accuracy was 70.21 ± 5.72%, and it is clear that the classification accuracy covered the full range from chance-level performance (50%) to complete control (100%). When the classification accuracy was higher than 75%, this set of data was considered to have better MI-BCI performance, referred to as the “high group”, and the remaining data belonged to “low group”.

### 3.2. Relationship between Microstate Feature Parameters and Subjects’ MI-BCI Performance

The four microstate maps obtained by clustering the resting-state data of all subjects were shown in Figure 3b, which were consistent with the four microstates studied in Khanna et al. [22]. The four microstates were labeled MS1, MS2, MS3, and MS4, respectively, and microstate feature parameters (mean duration, occurrence per second, time coverage ratio, and transition probability) were calculated. The Pearson correlation coefficient between microstate feature parameters and the MI-BCI classification accuracy of 168 experimental data sets were calculated, and the correlation coefficient results are shown in Table 1. The MS1 mean duration, occurrence per second, time coverage ratio and transition probability were all negatively correlated with the subjects’ MI-BCI performance, and the MS1 occurrence per second was significantly correlated with the correlation coefficient r=−0.544(p<0.001). The MS3 mean duration, occurrence per second, time coverage ratio, and transition probability were all positively correlated with the subjects’ MI-BCI performance, and the MS3 mean duration was significantly correlated with the correlation coefficient r=−0.593(p<0.001). The MS2 mean duration, occurrence per second, and time coverage ratio were all negatively correlated with the subjects’ MI-BCI performance, and some of the transition probabilities were positively correlated. In contrast, some of the transition probabilities were negatively correlated with the subjects’ MI-BCI performance. The MS4 feature parameters were similar to those of MS2. In conclusion, the MS1 occurrence per second and MS3 mean duration were chosen to predict the subjects’ MI-BCI performance.

**Figure 5:**
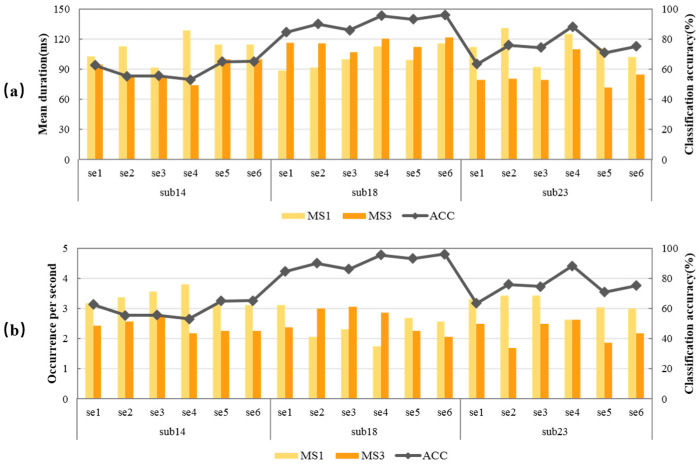
Relationship between classification accuracy and MS1, MS3 of the three representative subjects. (**a**) MS1 mean duration and MS3 mean duration; (**b**) MS1 occurrence per second and MS3 occurrence per second.

Three subjects with different MI-BCI performances were selected to illustrate the relationship between classification accuracy and microstate feature parameters. The classification accuracy of all sessions of subject 14 was lower than 75%, which all belong to the “low group”. All sessions of subject 18 were higher than 75%, belonging to the “high group”. Some sessions of subject 23 belong to the “low group”, and some to the “high group”. The relationship between classification accuracy and MS1 and MS3 of the three subjects were statistically analyzed, and the results are shown in Figure 5. The dark orange column represents MS3, the light orange column represents MS1, and the gray line represents classification accuracy.

In Figure 5a, the change trend of the dark orange column was very similar to that of the gray line, indicating that the classification accuracy was extremely related to MS3 mean duration. The higher the MS3 mean duration, the better the MI-BCI performance. In Figure 5b, the change trend of the light orange column was opposite that of the gray line, indicating that the classification accuracy was extremely related to MS1 occurrence per second. The lower MS1 occurrence per second, the better the MI-BCI performance. The negative correlation of MS1 occurrence per second (r=−0.820,p<0.001) and the positive correlation of MS3 mean duration (r=0.800,p<0.001) existed in all three subjects with different MI-BCI performance, which indicated the relationship between MS1 and MS3 and subjects’ MI-BCI performance was universal.

### 3.3. Microstate Prediction Performance

The normalized MS1 occurrence per second and the normalized MS3 mean duration of the resting-state EEG from MI experimental data were used to predict subjects’ MI-BCI performance, which was named microstate predictor. The predictor was used to identify whether the data belonged to the “high group” or “low group”. A linear discriminant analysis (LDA) classifier was used to build the prediction model, and the feature dimension is 2. In the prediction, a leave-one-out cross-validation scheme was used separately for every session, and the number of subjects in the “high group” and “low group” in each session is shown in Table 2.

**Figure 6:**
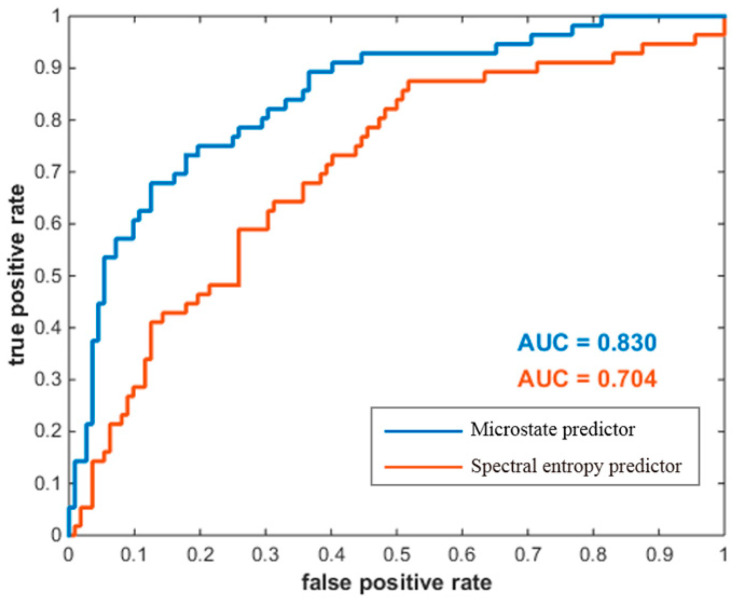
ROC curves for the two predictors in classifying the two groups of MI-BCI users. The horizontal coordinate denotes the false-positive rate, and the vertical coordinate denotes the true-positive rate. The AUC area under curve is the basis for judging the prediction performance.

A receiver operating characteristic (ROC) analysis was first conducted to evaluate the classification capability of the proposed microstate predictor for all data, where the “high group” and the “low group” were defined as positives and negatives, respectively. As shown in Figure 6, microstate predictor’s prediction performance was compared with spectral entropy predictor. The resulting area under curve (AUC) was 0.830 for the microstate predictor, and 0.704 for the spectral entropy predictor.

## 4. Discussion

### 4.1. Subjects’ MI-BCI Performance

The high interpretability of the regression model indicated that the fluctuation of MI-BCI performances could be explained by MS1 occurrence per second (r=−0.820,p<0.001) and MS3 mean duration (r=0.800,p<0.001). In addition, the microstate potential topographic maps of MS1 and MS3 are related to the physiological characteristics of the brain. Interpreted from the microstate potential topographic maps, in the map of MS3, the energy was higher in the anterior frontal region, and lower in the occipital region. While the primary motor cortex in the frontal cortical region is involved in the planning, control, and coordination of movements, the increase in its energy may represent the enhancement of the subject’s MI-BCI performance. In the map of MS1, the energy was higher in the right region, and lower in the left region. The decrease in MS1 occurrence per second in the resting-state EEG was a characteristic of the subjects’ better MI-BCI performance, which may be related to the fact that the subjects were all right-handed.

In a joint study of resting-state EEG microstates and fMRI, Michel et al. showed that microstate C is associated with a resting-state network responsible for integrating interoceptive information [25]. During slow-wave sleep, the executive control network overlapped the fMRI deactivation of microstate C [24]. In this paper, the increase in MS3 (microstate C) mean duration in the resting-state EEG before the MI task was another characteristic of the subjects’ better MI-BCI performance, which can be understood as the emergence of executive control networks, and the enhancement of the brain’s autonomic neural processing ability is related to the subjects’ MI-BCI performance [34,35].

### 4.2. Number of Microstate Maps

In order to obtain the best clustering effect, it is crucial to determine the number of microstate maps. Therefore, we need to measure the ability of different numbers of microstates to fit EEG data. First, we calculate three measures of fit: global explained variance (GEV), cross-validation criterion (CV), and dispersion. GEV measures the correlation between the EEG signal sample and the microstate that this sample belongs to. It can be calculated as follows:

(3) $$C_{u,v}=\frac{\sum_{i=1}^{n}u_{i}\cdot v_{i}}{\sqrt{\sum_{i=1}^{n}u_{i}^{2}}\cdot \sqrt{\sum_{i=1}^{n}v_{i}^{2}}}$$

(4) $${GEV}_{n}={C_{x_{n}a_{n}}}^{2}\frac{{{GFP}_{n}}^{2}}{\sum_{n^{\prime }}^{N}{GFP}_{n^{\prime }}^{2}}$$

where $C_{u,v}$ represents the squared correlation between two individual topographies *u* and *v*, $u_{i}$ represents the voltage of channel i on the topography *u*, and $v_{i}$ represents the voltage of channel i on the topography *v*. Then, GEV can be defined as the squared correlation between the EEG sample and its microstate map, weighted by the proportion of the EEG sample to the total square of GFP. Where ${GFP}_{n}$ represents the global field power of EEG for the $n^{\prime }$th time sample, N is the number of all EEG data sample points amount.

A higher GEV value represents a better fitting effect. The GEV value is the mean of the sum of the GEV values of the all EEG data sample points:

(5) $$GEV=\frac{1}{N}\sum_{n^{\prime }}^{N}{GEV}_{n^{\prime }}$$

The cross-validation criterion (CV) is a fitting parameter related to residual noise, so we need to obtain a low CV value. It can be defined as follows:

(6) $$V={\hat{\sigma }}^{2}\cdot {\left(\frac{C-1}{C-K-1}\right)}^{2}$$

where C is number of the channel, *K* is the number of microstates, and ${\hat{\sigma }}^{2}$ is the value of residual noise variance. ${\hat{\sigma }}^{2}$ can be calculated:

(7) $${\hat{\sigma }}^{2}=\frac{\sum_{n}^{N}x_{n}^{T}x_{n}-{\left(a_{n}^{T}x_{n}\right)}^{2}}{N\left(C-1\right)}$$

where N represents the number of EEG sample points, $x_{n}$ represents the topography of the *n*th EEG sample point, and $a_{n}$ represents the topography of the microstate to which the *n*th EEG sample point belongs.

Dispersion can estimate the mean distance between the EEG samples of the same cluster. For the data with *K* microstates, the dispersion $D_{k}$ is the sum of squares between sample points in each microstate category:

(8) $$S_{k}=\sum_{n}^{N}\sum_{n^{\prime }}^{N}{\left\|x_{n}-x_{n^{\prime }}\right\|}^{2},for\;l_{n}=k⋀l_{n^{\prime }}=k$$

(9) $$D_{k}=\sum_{k}^{K}\frac{S_{k}}{2N_{k}}$$

where $N_{k}$ is the number of sample points belonging to the class *k* microstate, $x_{n}$ and $x_{n^{\prime }}$ are two individual samples belonging to the class *k* microstate. $D_{k}$ can be seen as a measure of error, the lower the better.

**Figure 7:**
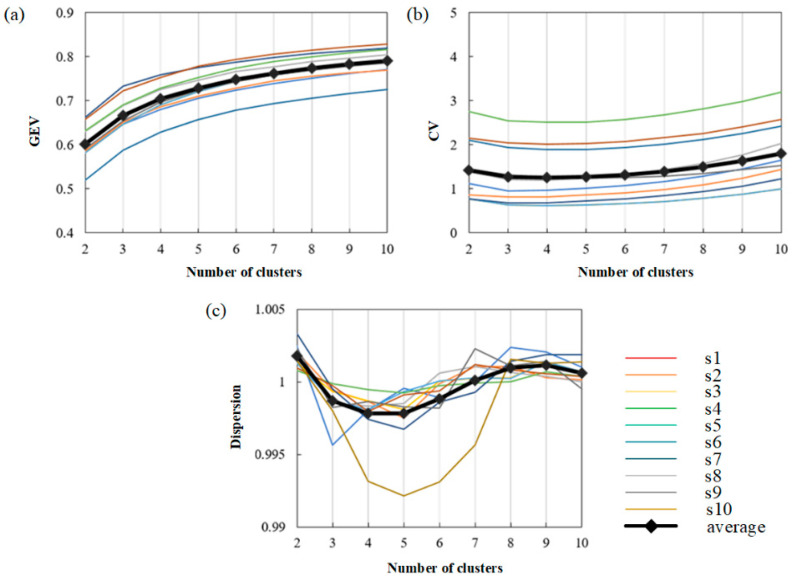
The ability to fit MI-EEG signals under different cluster numbers. (**a**) The value of GEV with different cluster numbers in 10 subjects. (**b**) The value of CV with different cluster numbers 10 subjects. (**c**) The value of dispersion with different cluster numbers in 10 subjects.

Then, ten subjects were selected to discuss the changes in three measures under different clustering numbers (2–10). The results are shown in Figure 7.

Finally, the values of three measures on the average level and the trend of the average curve are observed. As shown in Figure 7a, the curve trend of each subject is basically the same. It can be seen that the GEV value increases with the increase in the number of clusters, showing a positive correlation with the number of clusters. Figure 7b shows that the value of CV firstly decreases and then increases with the increase in the cluster number. The minimum value of CV is obtained when the cluster number is 4, which means that the four clusters will obtain the best-fitting result. In Figure 7c, we can see that with the increase in the number of clusters, the dispersion decreases first, then increases, and then decreases. The dispersion is low when the number of clusters is four or five. After comprehensive consideration, the number of clusters used in this paper is 4.

### 4.3. Microstate Predictor and Spectral Entropy Predictor

Compared with the spectral entropy predictor, the microstate predictor achieved better prediction performance. Although the performance of the spectral entropy predictor is not good enough, it adopted a statistical method to represent the power spectral density, which is robust enough to offset the negative impact of noise on prediction. Differing from the spectral entropy predictor, which calculated the probability distribution information of the power spectrum using single-channel EEG data, the microstate predictor considers multi-channel information, which can extract a wealth of information in both spatial and temporal domains. The microstate predictor can reveal how various brain regions communicate at different time points. This may provide a new insight into MI-BCI performance prediction. Therefore, compared with other predictors, the microstate predictor, which contains more information, may be more efficient and stable in analyzing differences in motor imagery performance between subjects.

### 4.4. Cross-Session Analysis of the Subject’s MI-BCI Performance Prediction

**Figure 8:**
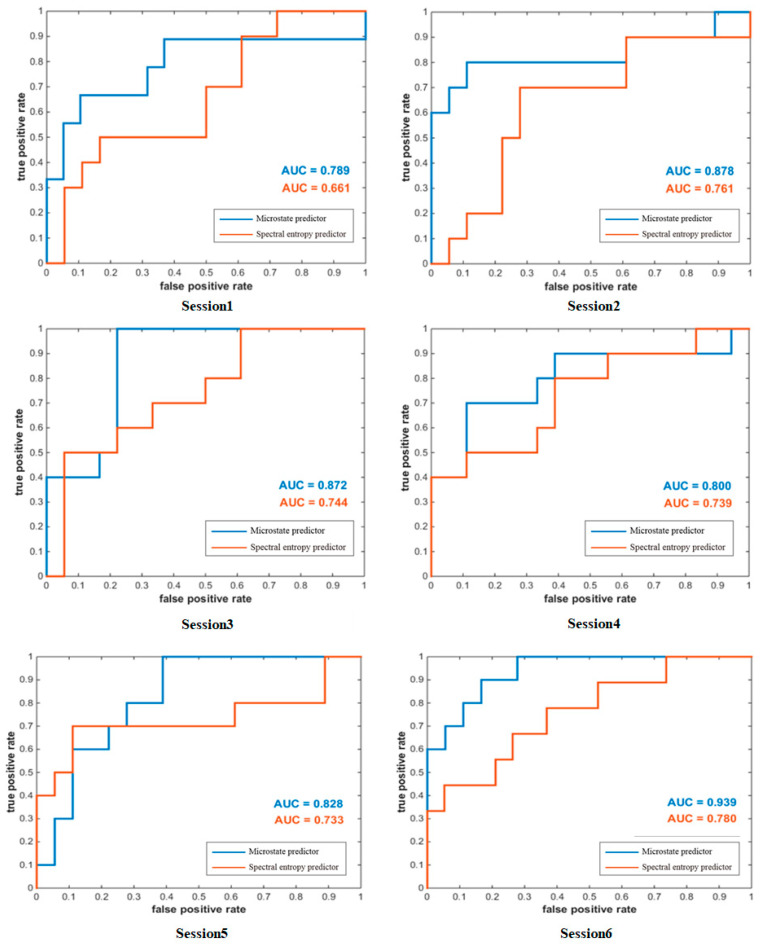
ROC curves for the two predictors in single session.

The difference in subjects’ MI-BCI performance not only exists between subjects, but also between experiments performed by the same subject in different periods. Using datasets collected from other times can better reflect the stability of the microstate predictor. A cross-session analysis of the subject’s MI-BCI performance according to the microstate predictor and spectral entropy predictor is shown in Figure 8. The AUC of the microstate predictor is higher than that of the spectral entropy predictor at both the single-session level and average level. Moreover, the AUC values fluctuated from session 1 to session 6, and are both the lowest in session 1 and the highest in session 6. This may be related to the motor imagery ability of subjects, which was gradually improved with the experiment.

### 4.5. Future Study

This work can be applied to the subject selection, evaluation of the training effect of the subjects, and the evaluation of the effect of rehabilitation training for patients. There are two limitations in the present work. First, the selection of the number of microstates and the number of channels affect the microstate predictor; thus, it is necessary to discuss our method’s performance in different channels and microstates. Second, another validation dataset is needed to verify the effectiveness of our approach.

## 5. Conclusions

In this paper, an EEG microstate analysis was used for subjects’ MI-BCI performance. The negative correlation of MS1 occurrence per second and the positive correlation of MS3 mean duration with subjects’ MI-BCI performance was explored, and the proposed predictor based on microstate feature parameters provides outstanding classification capability for the “high group” and “low group”. The EEG microstate provides an effective new way to analyze the differences in subjects’ MI-BCI performance.

## Figures and Tables

**Table 1:** Pearson correlation coefficient between microstate feature parameters and MI-BCI classification accuracy of 168 sets of data. Significant results (*p* < 0.001) are marked in bold. Stars correspond to the significance levels (‘**’ means *p* < 0.001, ‘*’ means 0.01 < *p* < 0.05).

Parameters	MS1		MS2		MS3		MS4	
	*r*	*p*	*r*	*p*	*r*	*p*	*r*	*p*
Duration	−0.388	0.025 *	−0.036	0.482	0.593	<0.001 **	−0.057	0.464
Occurrence	−0.544	<0.001 **	−0.022	0.575	0.263	0.032 *	−0.035	0.483
Coverage	−0.141	0.067	−0.093	0.240	0.176	0.053	−0.071	0.355
TP1	/	/	−0.204	0.041 *	−0.055	0.474	−0.090	0.245
TP2	0.004	0.961	/	/	−0.004	0.962	−0.218	0.039 *
TP3	0.146	0.059	0.495	<0.001 **	/	/	0.463	<0.001 **
TP4	−0.143	0.064	−0.077	0.324	0.057	0.464	/	/

**Table 2:** The number of subjects in the “high group” and “low group” in each session.

	High Group	Low Group
Session 1	7	21
Session 2	10	18
Session 3	9	19
Session 4	10	18
Session 5	11	17
Session 6	11	17

## Data Availability

The raw data supporting the conclusions of this article will be made available by the authors, without undue reservation.
